# Supplementary material for: D-α-Tocopherol-Based Micelles for Successful Encapsulation of Retinoic Acid
Source: Pharmaceuticals (Basel). 2021 Mar 4;14(3):212. doi: 10.3390/ph14030212 (PMC7999664; doi:10.3390/ph14030212)
Supplement: Supplementary file 1 [file pharmaceuticals-14-00212-s001.pdf]

pharmaceuticals

Supplementary Information

D-α-tocopherol-Based Polymeric Micelles for Successful Improving Encapsulation of Retinoic Acid

Guendalina Zuccari[1,*], Sara Baldassari[1], Silvana Alfei[1], Barbara Marengo[2], Giulia Elda Valenti[2], Cinzia Domenicotti[2], Giorgia Ailuno[1], Carla Villa[1], Leonardo Marchitto[3], and Gabriele Caviglioli[1]

[1] Department of Pharmacy (DiFAR), University of Genoa, Viale Cembrano 4, I-16148, Genova, Italy, zuccari@difar.unige.it, baldassari@difar.unige.it, alfei@difar.unige.it, ailuno@difar.unige.it, villa@difar.unige.it, caviglioli@difar.unige.it
[2] Department of Experimental Medicine - DIMES, University of Genoa, Via Alberti L.B. 2 I- 16132 Genoa, Italy; barbara.marengo@unige.it; giuliaelda.valenti@edu.unige.it; cinzia.domenicotti@unige.it
[3] Department of Sciences for the Quality of Life, University of Bologna, Corso D'Augusto 237, Rimini, Italy, leonardo.marchitto@unibo.it
* Correspondence: zuccari@difar.unige.it (G.Z.)

Section S1. Figure S1-S15.

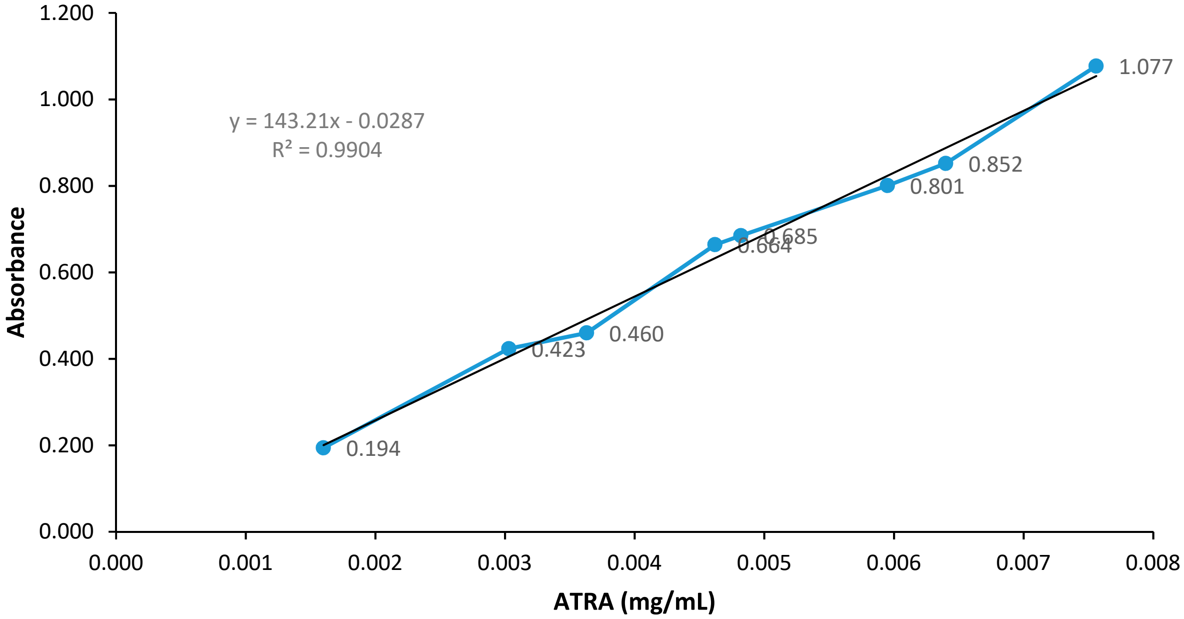

Figure S1. ATRA calibration curve in methanol at λ_{max} = 340 nm using an UV-Vis spectrophotometer within a range 5.33 x 10^{-3} – 2.13 x 10^{-2} μM.

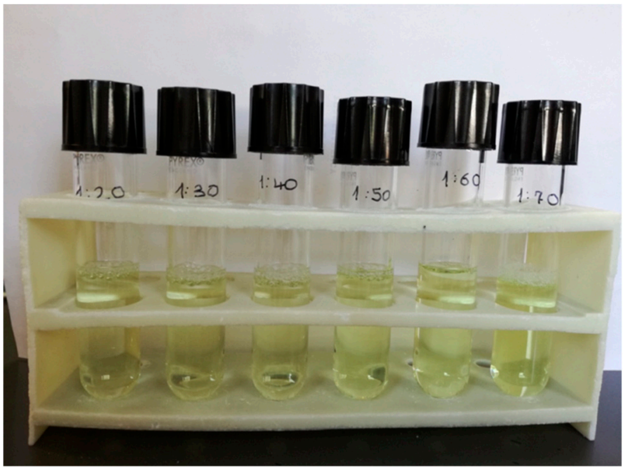

Figure S2. ATRA-TPGS colloidal dispersions obtained at different ATRA:TPGS ratios (w:w) by solvent casting method.

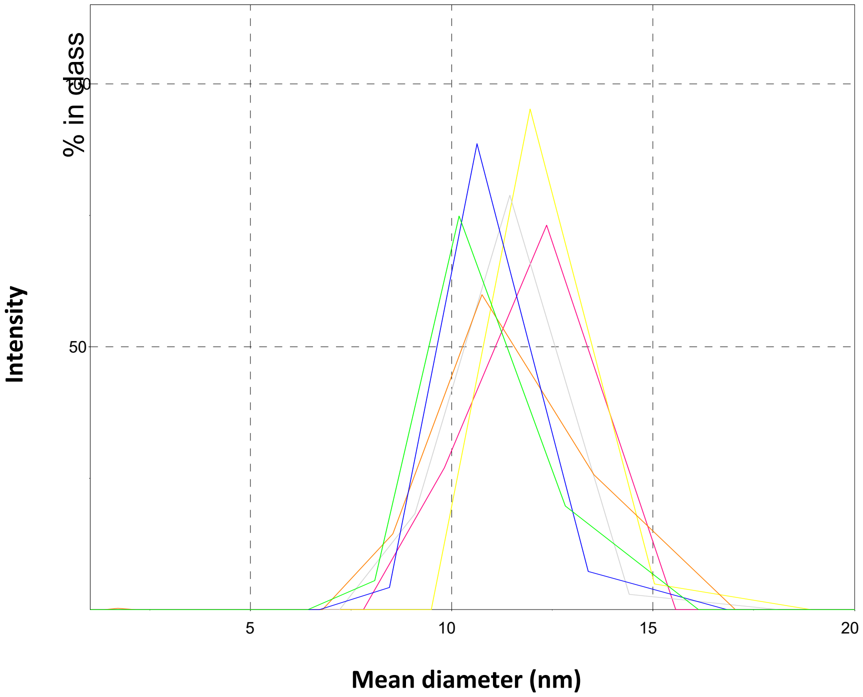

Figure S3. Representative size distribution of different freshly prepared ATRA-TPGS formulations. Green line corresponds to 1:70 ratio, blue line to 1:60 ratio, orange line to 1:50 ratio, grey line to 1:40 ratio, yellow line to 1:30 ratio and red line to 1:20 ratio.

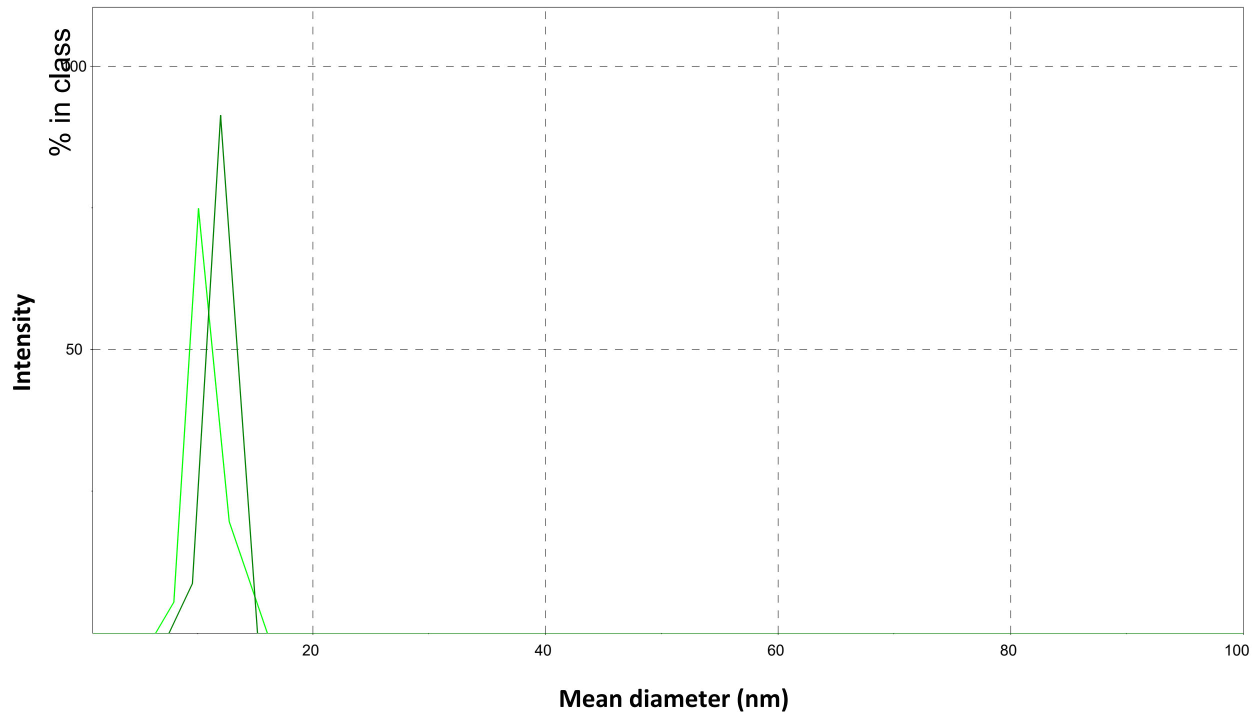

Figure S4. Representative size distribution of ATRA-TPGS formulation at 1:50 ratio. Light green line corresponds to a freshly prepared micellar dispersion, and green line to micelles reconstituted in water from lyophilized powder.

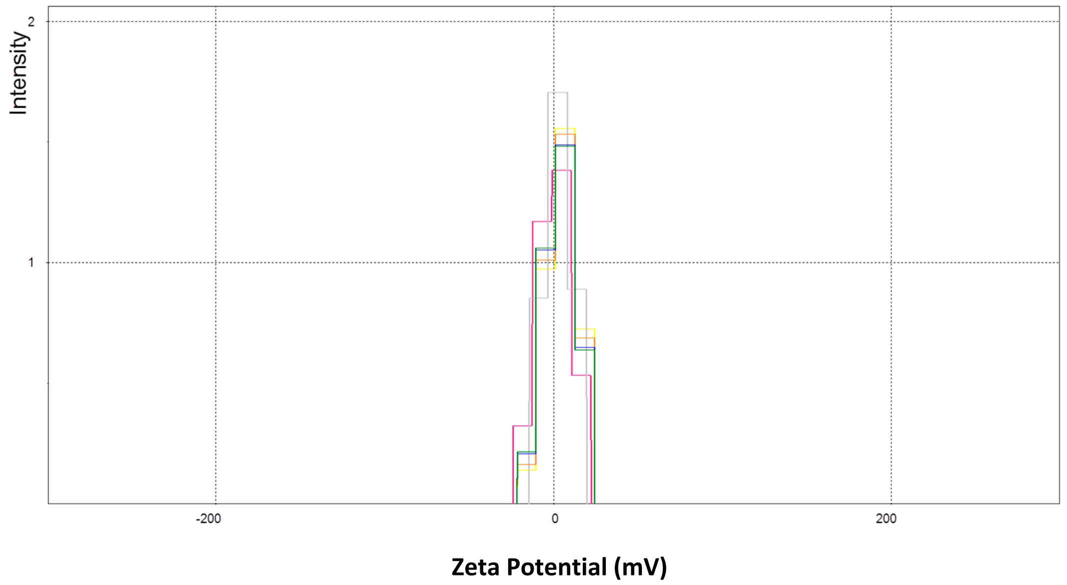

Figure S5. Representative Z potentials distribution of different ATRA-TPGS formulations measured in HEPES buffer (pH 7.4). Green line corresponds to 1:70 ratio, blue line to ratio 1:60, orange line to 1:50 ratio, grey line to 1:40 ratio, yellow line to 1:30 ratio and red line to 1:20 ratio.

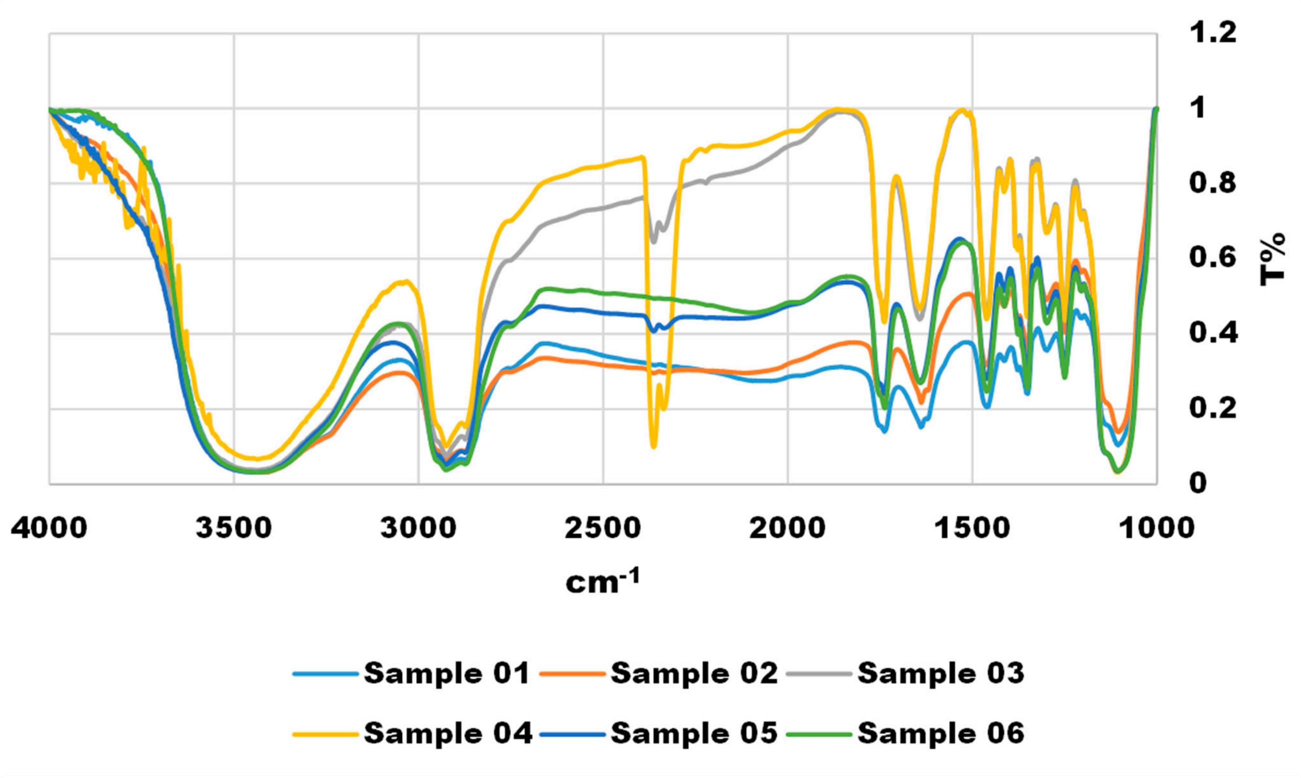

Figure S6. FTIR spectra of ATRA-TPGS formulations obtained using ATRA:TPGS ratios of 1:20, 1:30, 1:40, 1:50, 1:60 and 1:70 and named Sample 01, 02, 03, 04, 05 and 06 respectively.

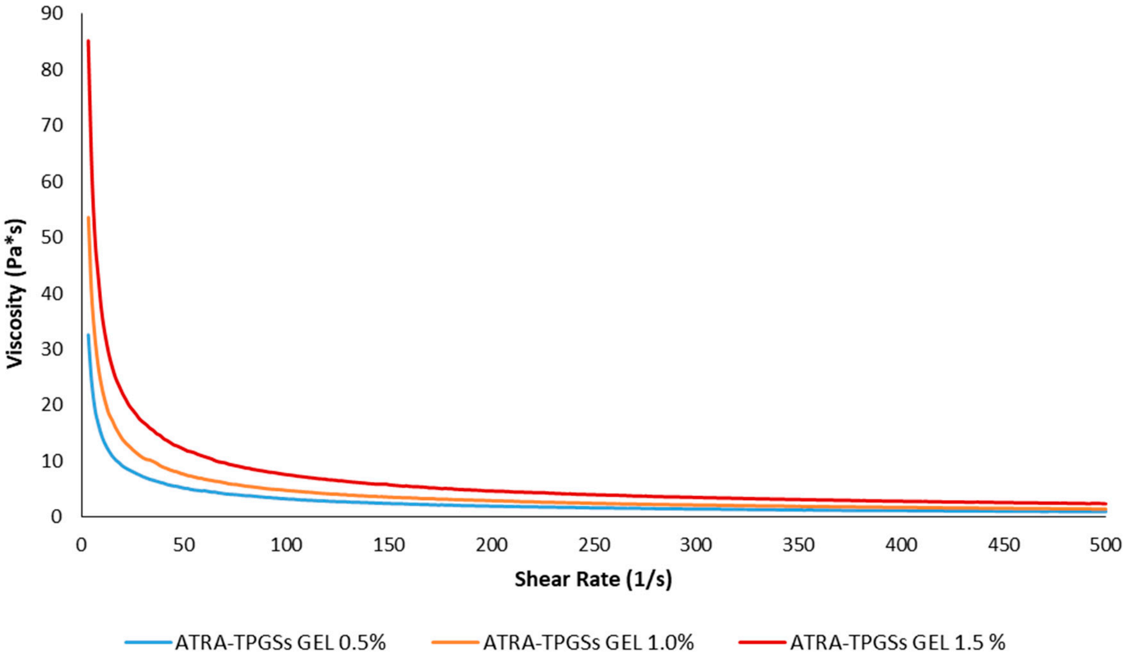

Figure S7. Viscosity curves of ATRA-TPGSs loaded gels at 0.5%, 1.0% and 1.5% (w/w) Carbopol® 980 concentrations, recorded at 25 °C using a concentric cylinder viscometer equipped with a Z4 probe.

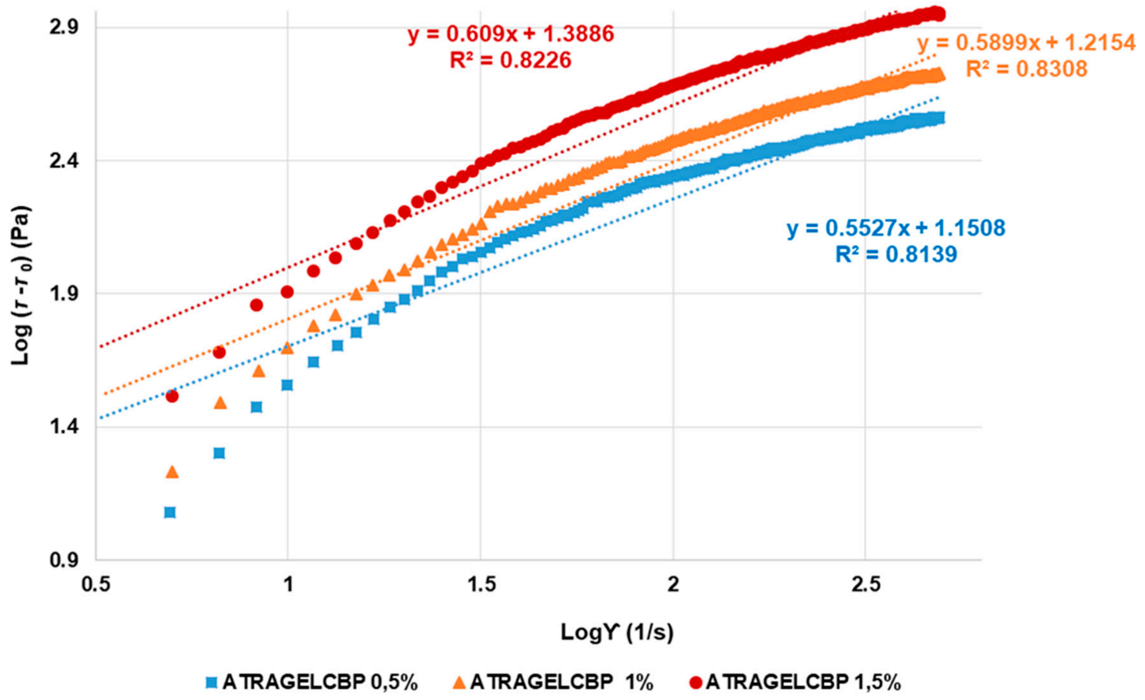

Figure S8. Hershel-Buckley rheograms of ATRA-TPGSs loaded gels at 0.5%, 1.0% and 1.5% (w/w) Carbopol® 980 concentrations.

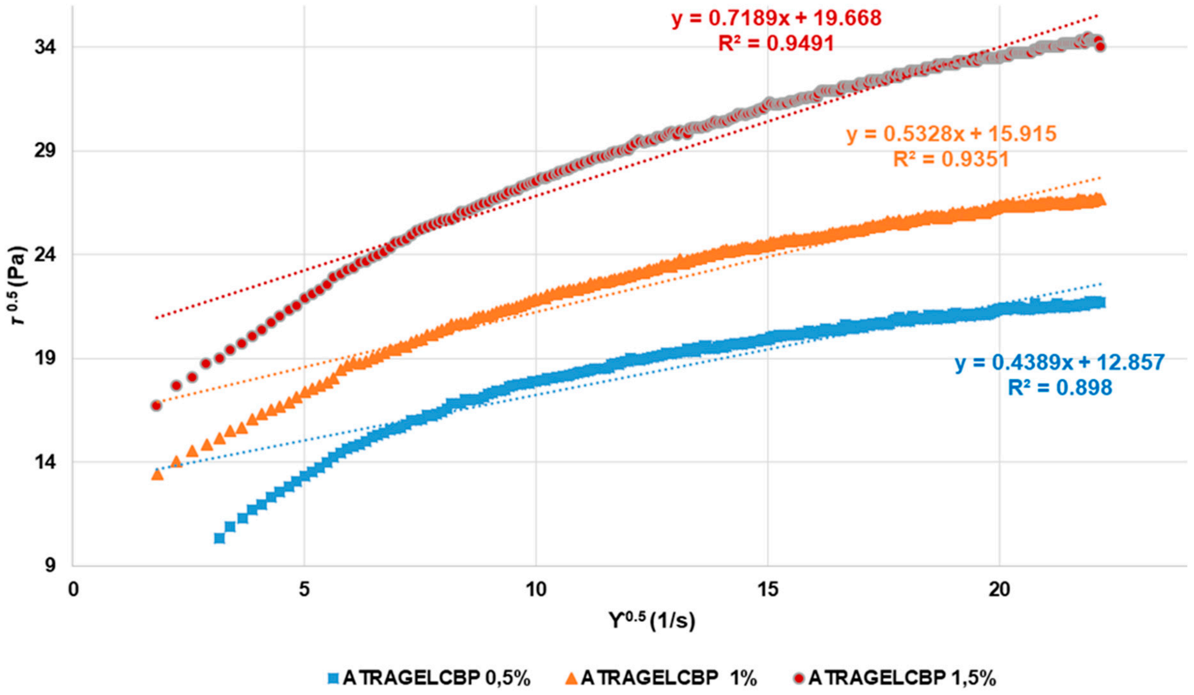

Figure S9. Casson rheograms of ATRA-TPGSs loaded gels at 0.5%, 1.0% and 1.5% (w/w) Carbopol® 980 concentrations.

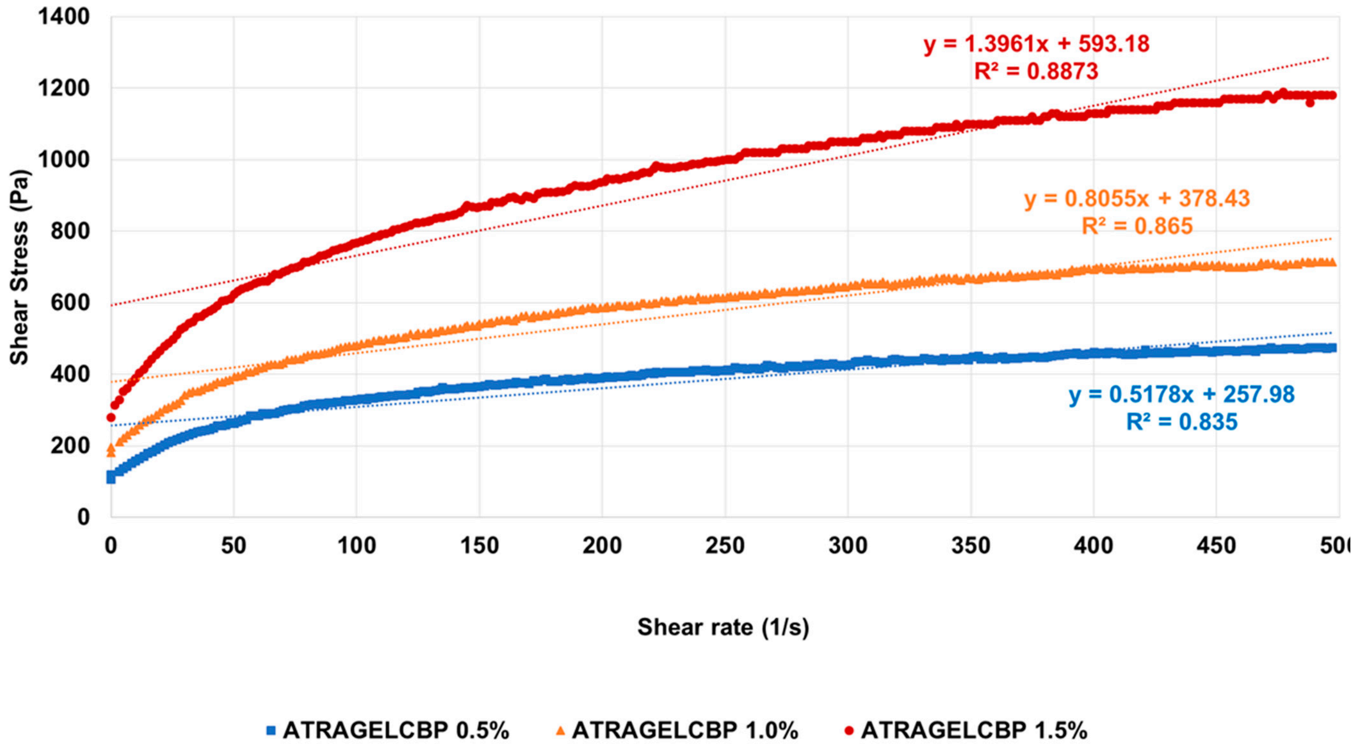

Figure S10. Bingham Plastic rheograms of ATRA-TPGSs loaded gels at 0.5%, 1.0% and 1.5% (w/w) Carbopol® 980 concentrations.

Section 2. Table S1.

Table S1. Models parameters, i.e. *n* and *n*H, k and kH, τ_{oc}, η_{cp}, τ_o and η_p.

**Model**	***n***	**k**	τ_{oH}	τ_{oc}	η_{cp}	τ_o	η_p
Power Law	0.2877 [1]	83.04					
	0.2866 [2]	124.94					
	0.3015 [3]	187.20					
Herschel-Bulkley	0.5527 [1]	14.15	107				
	0.5899 [2]	16.42	180				
	0.6090 [3]	24.47	280				
Casson				165.30 [1]	0.19		
				253.61 [2]	0.28		
				386.83 [3]	0.52		
Bingham Plastic						257.98 [1]	0.5178
						378.43 [2]	0.8055
						593.18 [3]	1.3961

[1] ATRA-TPGSs loaded gel with Carbopol [1] 0.5%, [2] 1.0%, [3] 1.5% (w/w).
